# First-line Eradication of Helicobacter pylori Infection with High-Dose Amoxicillin and Vonoprazan: A Systematic Review and Meta-analysis

**DOI:** 10.5152/tjg.2025.24371

**Published:** 2025-06-16

**Authors:** Hongxiu Yu, Zhengwen Zhou, Zhi Liu

**Affiliations:** 1 Department of Pharmacy, Panjiang Coal and Electricity Group Hospital, Guizhou, China; 2 Dali University College of Pharmacy, Yunnan, China; 3 Department of Pathology, Affiliated Hospital of Guizhou Medical University, Guizhou, China

**Keywords:** Dual therapy, *Helicobacter pylori*, high-dose amoxicillin, meta-analysis, vonoprazan

## Abstract

**Background/Aims:**

Due to increasing *Helicobacter pylori* resistance, the effectiveness of proton pump inhibitor-based regimens has diminished. There is a need to assess the high-dose amoxicillin (≥3 g/day) and vonoprazan dual therapy through meta-analysis to determine if it is more effective than previous protocols.

**Materials and Methods:**

A computer search of PubMed, Web of Science, Cochrane Library, Embase, and ClinicalTrials.gov was carried out to identify randomized controlled trials of massive dose amoxicillin and vonoprazan double treatment for *H*.* pylori* infection, up to June 2023. The primary outcome measures were the eradication rate and adverse event rate.

**Results:**

A total of 7 studies encompassing 2601 patients were analyzed. The eradication rate of the high-dose dual group has no statistically significant differences compared to the control group (non-high-dose amoxicillin combined with vonoprazan) in intention-to-treat (ITT) [OR=1.12, 95% CI (0.85, 1.48), *P* = .42] and per-protocol (PP) analysis [OR=1.08, 95% CI (0.74, 1.58), *P* = .69]. The incidence of adverse events [OR=0.63, 95% CI (0.45, 0.88), *P* = .007] was significantly reduced in the dual treatment group. Subgroup analysis revealed that the eradication rate in China [OR=1.34, 95% CI (1.01, 1.87), *P* = .04] was higher for the high-dose dual group compared with the control group in ITT analysis.

**Conclusion:**

The eradication rate of high-dose amoxicillin and vonoprazan dual regimen is similar to that of previous regimens, and the adverse event rate is significantly lower.

**Main Points**

- High eradication rate: High-dose amoxicillin combined with vonoprazan showed a high eradication rate against *Helicobacter pylori*.
- Good safety: The combination of drugs is safe and the adverse reactions are acceptable.
- Good compliance: The patient’s compliance with the treatment plan is high, which is conducive to the development of treatment.
- Comparative advantage: Compared with some traditional schemes, it has certain advantages.
- Widely applicable: Suitable for a variety of *H. pylori* infection populations.

## Introduction

*Helicobacter pylori* (*H*.* pylori*) is a spiral-flagellated, microaerobic, gram-negative bacterium.^1^ Infection with *H*.* pylori* has been linked to a range of gastrointestinal disorders, such as chronic atrophic gastritis, peptic ulcers, vitamin B_12_ deficiency, and iron-deficiency anemia.^2^^-^^4^ Classified as an infectious disease,^5^ *H*.* pylori* infection has profound effects on gastric mucosal health. Importantly, its eradication can notably diminish the risk of developing gastrointestinal diseases.

Global variations in the epidemiology of *H*.* pylori* demand regionally tailored first-line eradication regimens. In regions where clarithromycin resistance remains low, triple therapy incorporating proton pump inhibitors (PPIs) remains first line. Conversely, in areas where clarithromycin resistance exceeds 15%, a bismuth-containing quadruple therapy is recommended. This recommendation is echoed in the recent Maastricht-6 consensus, which advocates for the use of bismuth-based quadruple therapy.^6^^,^^7^ Nevertheless, the escalating rates of antibiotic resistance pose a significant challenge, as *H*.* pylori* increasingly develops dual or even multiple resistances to clarithromycin, metronidazole, levofloxacin, and other antibiotics.^8^ Consequently, achieving optimal eradication rates with these standard regimens has become increasingly difficult.

Amoxicillin is one of the first-line antibiotics for *H*.* pylori* eradication therapy, which has a better killing effect on *H*.* pylori* in reproduction. The high pH state in the stomach provided by acid inhibitors can induce *H*.* pylori* to enter the reproduction period and increase the stability of amoxicillin.^9^^,^^10^ Proton pump inhibitors need to be activated in an acidic environment to play a role, and the half-life is short. Therefore, it usually leads to insufficient control of gastric pH, which may lead to treatment failure, and its efficacy is also affected by genetic polymorphism.^11^^,^^12^ Although PPIs are effective antacids and only 1 % ~ 3 % of short-term adverse reactions may occur during treatment, long-term use of PPIs can lead to a higher risk of adverse reactions, especially in the elderly. The risk of adverse reactions such as fractures and kidney diseases during treatment may also increase the risk of death from infectious pneumonia and other related adverse outcomes.^13^^,^^14^ Vonoprazan, a potassium-competitive acid blocker developed by Takeda,^15^ inhibits acid by reversibly competing with potassium ions at the H^+^, K^+^-ATPase-binding sites.^16^ This compound was approved for market sale in Japan in 2015 and it was recommended as a first-line treatment for *H*.* pylori* eradication by the Japanese *H*.* pylori* infection treatment guidelines in 2016.^17^ Jenkins H et al’s^18^ study demonstrated that 40 mg/day of vonoprazan maintained intragastric pH value greater than 5 for 98% of a 24-hour cycle after 7 days, providing significantly longer and more intense acid suppression than PPIs. Vonoprazan’s ability to maintain a prolonged high pH state in the stomach encourages *H*.* pylori* to enter a reproductive phase, potentially enhancing the bactericidal effect of antimicrobial drugs and improving *H*.* pylori* eradication efficacy.

The randomized controlled trial conducted by Sho Suzuki et al^19^ demonstrates that a combination therapy using low-dose amoxicillin (≤2 g/day) and vonoprazan achieved satisfactory eradication rates of *H*.* pylori* eradication.^20^ Furthermore, evidence suggests that a dual therapy based on PPIs combined with high-dose amoxicillin (≥3 g/day) is comparable to traditional treatment approaches.^21^ These findings prompt investigation into whether increasing the amoxicillin dosage within the dual therapy of amoxicillin and vonoprazan could lead to a higher eradication rate of *H*.* pylori*.

In the dual therapeutic approach that combines high-dose amoxicillin with vonoprazan, the recommended dosage of amoxicillin is set at ≥3 g/day,^22^ divided into 3-4 daily doses. This dosage corresponds with the pharmacokinetic/pharmacodynamic (PK/PD) profile of amoxicillin, indicating a potential increase in eradication effectiveness. Nevertheless, they require rigorous evaluation. Presently, there are no published meta-analyses specifically assessing the dual regimen consisting of high-dose amoxicillin paired with vonoprazan. The aim of this study was to compile data from randomized controlled trials (RCTs) and perform a meta-analysis to assess the efficacy and safety of this RCTs-based dual regimen as a first-line treatment for *H*.* pylori* eradication, thus offering evidence to inform clinical decisions.

## Materials and Methods

As this study exclusively involves secondary analysis of published literature without direct human subject participation, it is exempt from requiring ethics committee approval or informed consent. To ensure methodological rigor, the study protocol was prospectively registered with PROSPERO (CRD42023449161), adhering to PRISMA guidelines for systematic reviews.

### Search Strategy

A comprehensive systematic search was performed in 5 major international databases (PubMed, Web of Science, Cochrane Library, Embase, and ClinicalTrials.gov) through June 30, 2023, to identify RCTs investigating vonoprazan-amoxicillin dual therapy for *H*.* pylori* eradication. The search strategy utilized a combination of controlled vocabulary terms (MeSH/Emtree) and free-text keywords, including “high-dose amoxicillin” (3-4 g/day), “vonoprazan,” “pylori eradication,” and related variants. Full search syntax with Boolean operators is available in Supplementary File 1, adhering to PRISMA-Search guidelines.

### Inclusion and Exclusion Criteria

Inclusion criteria: (1) Study Design: Published RCTs with parallel-group design. (2) Subjects: Treatment-naïve adults (≥18 years) with confirmed *H*.* pylori* infection diagnosed by ≥2 validated methods (13C/14C-urea breath test, rapid urease test, histology, or culture). (3) Interventions: Vonoprazan (20 mg twice daily) combined with high-dose amoxicillin (≥3 g/day divided into 3 doses). (4) Control measures: Regimens endorsed by guidelines or consensus for first-line eradication therapy, or regimens with comparable eradication rates, encompassing PPI-based triple therapy, bismuth quadruple therapy, non-bismuth quadruple therapy, vonoprazan triple or quadruple therapy, and vonoprazan paired with low-dose amoxicillin (≤2 g/day). (5) Outcomes: Eradication and adverse event rates were the primary endpoints.

Exclusion criteria encompassed: (1) Non-randomized designs (e.g., retrospective studies, single-arm trials). (2) Regimens containing non-study antibiotics (e.g., clarithromycin, furazolidone, rifabutin). (3) Studies with incomplete outcome data or protocol deviations affecting validity.

### Data Extraction and Evaluation

Two independent researchers conducted data extraction and quality evaluation, cross-checking their results upon completion. In case of disagreement, they consulted a third researcher for resolution. To eliminate duplicates, EndNote software was employed, followed by a screening process of titles and abstracts to exclude irrelevant literature. The remaining studies underwent a thorough full-text review before final inclusion decisions were made. Data extraction was facilitated by a custom-designed Microsoft Excel spreadsheet, capturing key variables such as the first author, publication date, study areas, sample size, age, sex, random allocation method, *H*.* pylori* detection method, eradication duration, interventions, controls, eradication rate, and the incidence of adverse events. The quality of the RCTs was assessed using the Cochrane Collaboration’s Risk of Bias tool, as outlined in the Cochrane Handbook for Systematic Reviews of Interventions.

### Statistical Analysis

The meta-analysis was conducted using RevMan 5.4 software (Company; City, Country). Dichotomous variables were expressed as odds ratios (ORs), and both the combined effect size and its corresponding 95% CI were computed. Heterogeneity was assessed using the *I*^2^ test. In cases where statistical heterogeneity was not observed among the studies (*I*^2^ < 50%), the fixed-effects model was employed for analysis. Conversely, when heterogeneity was present, the random-effects model was utilized. Publication bias was evaluated through the generation of an inverted funnel plot. Statistical significance was determined at a *P*-value less than .05.

## Results

### Data Selection

**Figure 1.**
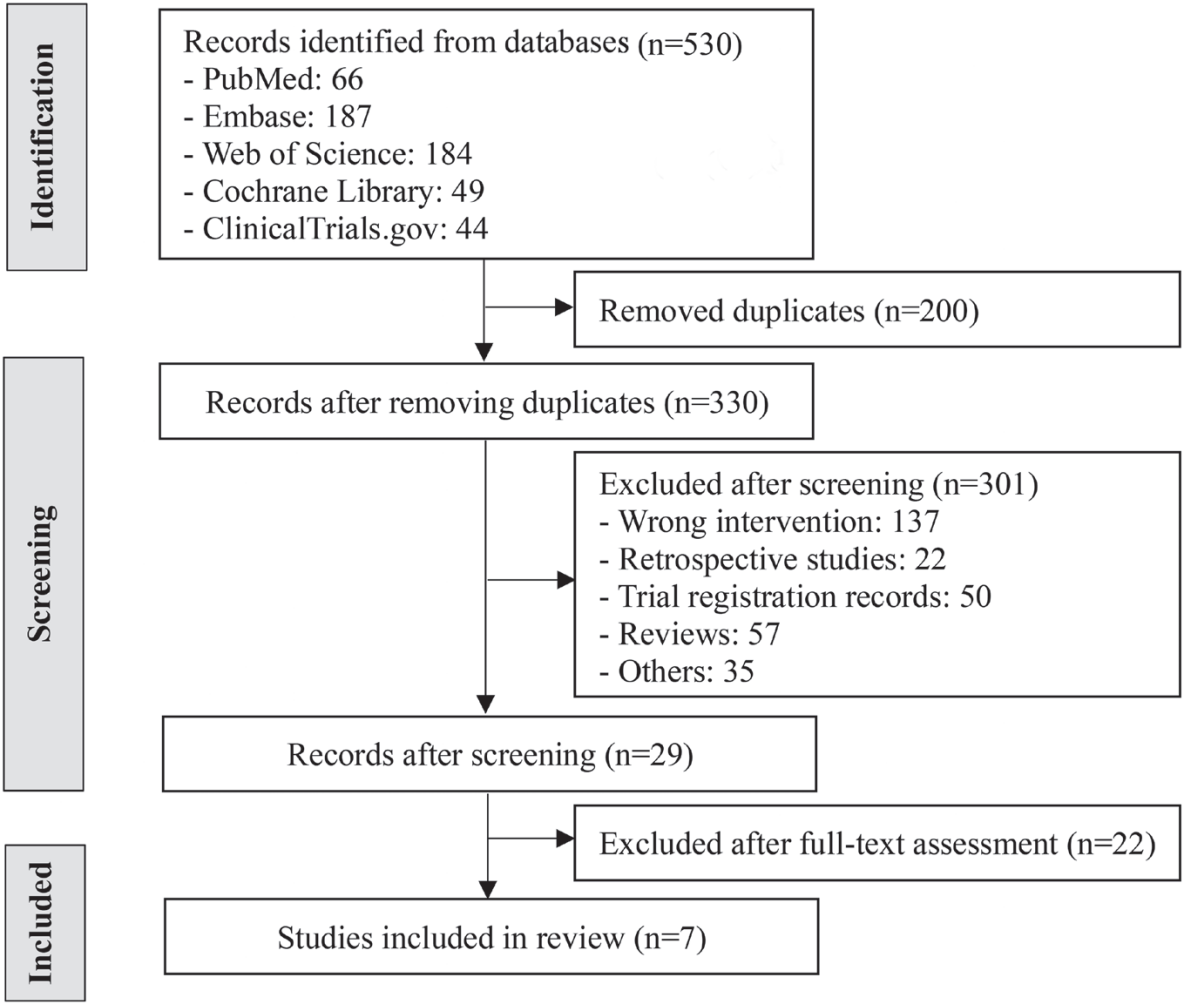
Literature screening flowchart.

A total of 530 literatures were retrieved from which 200 duplicates and 301 irrelevant articles, identified by title and abstract screening, were eliminated. After a full-text review of the remaining 29 articles, 7 RCTs were included. The screening process is illustrated in Figure 1.

### Study Characteristics

All studies were published in English between 2022 and 2023. Among these, 4 articles included 2 experimental groups.^23^^-^^26^ Data from each experimental group were extracted and compared separately to the control group. A total of 6 studies were conducted in China,^23^^,^^25^^-^^29^ while 1 study was conducted in the United States or Europe.^24^ These studies collectively involved 2601 participants. The basic characteristics encompassed in the study are summarized in Table 1. All studies documented the use of random allocation methods. Despite being open-label experiments, qualitative or quantitative laboratory testing for *H. pylori* suggested that blinding had minimal influence on assessing eradication success. Consequently, they were deemed to have a low risk of bias, and the comprehensive results of the risk-of-bias evaluation are presented in Table 2.

### Eradication Rate in Intention-To-Treat Analysis

**Figure 2.**
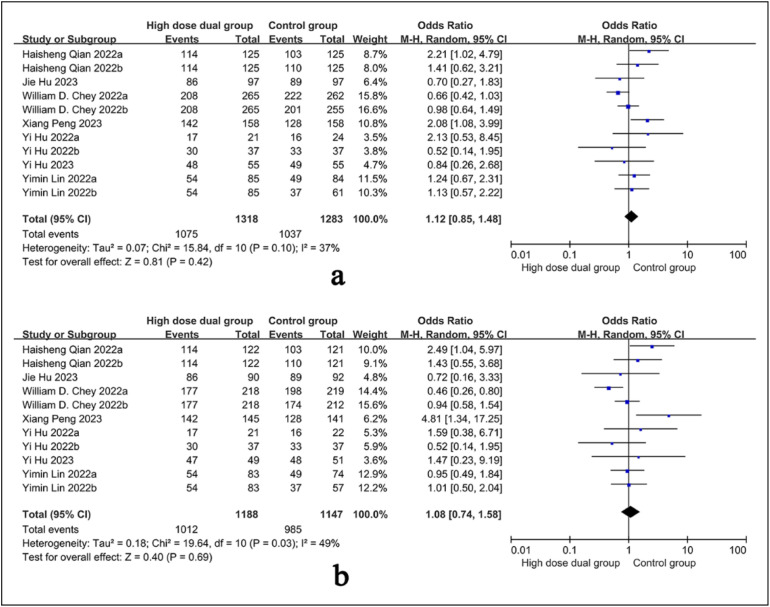
Forest plot of the eradication rate in intention-to-treat (A) and per-protocol (B) analysis.

All studies have consistently reported eradication rates in intention-to-treat (ITT) analyses, involving a collective total of 2601 patients. The pooled eradication rate in the high-dose combined therapy group stood at 81.6% (number cured / total number of patients). Heterogeneity test results were *P* = 0.1 and *I*^2^ = 37%, using a random effect model for combined effect size. The results indicated that the eradication rate of the high-dose dual regimen was higher than that of the control regimen [OR = 1.12, 95% CI (0.85, 1.48)], but the difference was not statistically significant (*P* = .42), as illustrated in Figure 2A.

### Eradication Rate in Per-Protocol

The eradication rate in PP analysis was reported across all studies, involving 2335 patients. The combined eradication rate for the high-dose dual therapy group was 85.2%. Heterogeneity test results were* P* = .03 and *I*^2^ = 49%, and the combined effect quantity adopts a random effect model. The results showed that the eradication rate of high-dose dual regimen was higher than that of the control regimen [OR = 1.08, 95% CI (0.74, 1.58)], but the difference was not statistically significant (*P* = .69), as shown in Figure 2B.

### Adverse Event Rate

**Figure 3.**
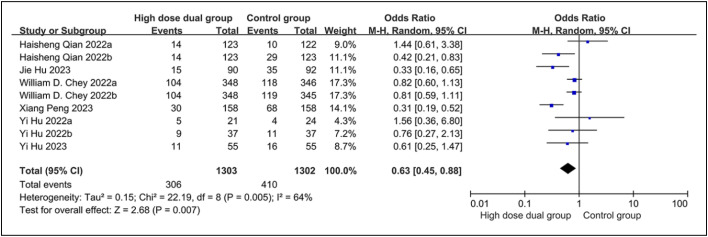
Forest plot of the adverse event rate.

Six studies reported the adverse event rate in 2605 patients. Heterogeneity test results were *P* = .005 and *I*^2^ = 64%, and a random-effects model was applied. The incidence of adverse events in the high-dose combination therapy group was lower than that in the control group [OR = 0.63, 95% CI (0.45, 0.88)], and this difference was statistically significant (*P* = .007), as shown in Figure 3.

### Subgroup Analysis of Eradication Rate

In ITT analysis, the eradication rate of the high-dose dual group in China [OR = 1.34, 95% CI (1.01, 1.78), *P* = .04] was significantly higher than that of the control group. However, there was no significant difference between the United States and Europe [OR = 0.81, 95% CI (0.55, 1.20), *P* = .29P]. The eradication rate for treatment durations of 7 days [OR = 1.26, 95% CI (0.82, 1.95), *P* = .29], 10 days [OR = 1.38, 95% CI (0.68, 2.79), *P* = .38], and 14 days [OR = 0.97, 95% CI (0.63, 1.47), *P* = .87] showed no significant differences.

In PP analysis, there was no significant difference in the eradication rate between the 2 regimens in China [OR = 1.31, 95% CI (0.89, 1.93), *P* = .17] or Europe and the United States [OR = 0.67, 95% CI (0.33, 1.35), *P* = .26]. Moreover, no significant differences in eradication rate were observed for treatment durations of 7 days [OR = 1.03, 95% CI (0.65, 1.62),*P* = .91], 10 days [OR = 1.38, 95% CI (0.61, 3.12), *P* = .44], and 14 days [OR = 1.02, 95% CI (0.49, 2.10), *P* = .96]. Subgroup analysis results suggested that regional differences might be the source of heterogeneity in the ITT analysis but not in the PP analysis. In addition, treatment duration did not contribute to heterogeneity in ITT or PP analysis. These results are presented in Table 3.

### Subgroup Analysis of Adverse Event Rate

In the subgroup analysis of study areas, the incidence of adverse events in the high-dose dual group was significantly lower than that in the control group in China [OR = 0.56, 95% CI (0.35, 0.89), *P* = .03]. In Europe and America, the incidence of adverse events in the high-dose double-drug group was lower than that in the control group [OR = 0.82, 95% CI (0.65, 1.02)], but the difference was not statistically significant (*P* = 0.08).

In the treatment duration subgroup, the results showed that there was no significant difference in the incidence of adverse events between the 2 regimens in the 10-day treatment group [OR = 0.74, 95% CI (0.35, 1.61), *P* = .45]. However, in the 14-day treatment group, the adverse event rate in the high-dose dual regimen [OR = 0.55, 95% CI (0.36, 0.85), *P* = .007] was significantly lower than in the control group. In addition, studying area and duration differences is not a source of heterogeneity in subgroup analysis, as indicated in Table 4.

### Sensitivity Analysis

When a study was excluded 1 at 1 time, there was no statistically significant difference in the change of the combined effect value.

### Publication Bias

**Figure 4.**
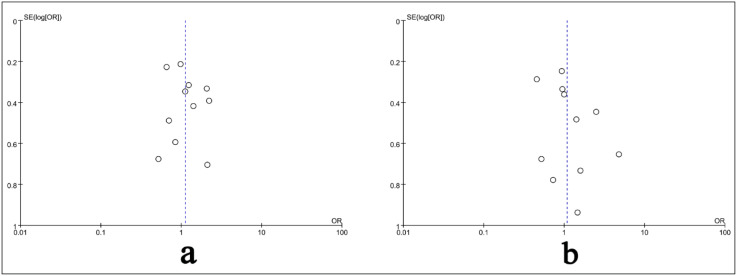
The funnel plot for the meta-analysis of the eradication rate.

The funnel plot for the meta-analysis of the eradication rate exhibited basic symmetry, suggesting a low likelihood of publication bias in the analysis of the total response rate, as depicted in Figure 4. Due to the limited number of studies included, publication bias has no obvious effect on the incidence of adverse events.

## Discussion

Since Marshall and Warren’s landmark identification of *H*.* pylori* in the gastric mucosal epithelium in 1983,^30^ its infection has affected over half of the global population, emerging as a widespread public health concern. *H*.* pylori* can acquire resistance through numerous mechanisms,^31^ resulting in a reduced eradication rate of conventional PPI-based therapies. Despite the escalating global resistance rates of clarithromycin and metronidazole, *H*.* pylori*’*s* resistance to amoxicillin remains remarkably low in most regions, suggesting that amoxicillin remains a cornerstone agent against *H*.* pylori*.

In this study, 7 RCTs were included to assess the effectiveness and safety of high-dose amoxicillin combined with vonoprazan for the treatment of *H*.* pylori* infection. The findings revealed that the eradication rates based on ITT and PP analyses were 81.6% and 85.2%, respectively. No statistically significant difference was observed in eradication rates between the high-dose dual therapy and the conventional quadruple therapy. This could be attributed to factors such as the duration of treatment and the limited antimicrobial spectrum of dual therapy. Furthermore, this investigation clarified that, when compared to other therapeutic approaches, compliance with the combination therapy of high-dose amoxicillin and vonoprazan did not exhibit notable variation. Importantly, the occurrence of adverse events linked to this combination therapy was notably lower compared to the previous regimen. These adverse events were minor and temporary, resolving swiftly after treatment discontinuation. The results of the subgroup analysis indicated that the effectiveness of this regimen might exhibit considerable variation across different countries. Although this therapy demonstrates a reduced incidence of adverse events and is non-inferior in terms of eradication rate compared to traditional regimens, careful consideration should be given to local drug resistance patterns when implementing this therapy as a first-line eradication treatment for *H*.* pylori*. This is due to the evident geographical disparities in eradication rates, potential side effects associated with high-dose amoxicillin, and the emergence of *H*.* pylori* resistance.

Amoxicillin, a time-dependent bactericidal agent, exerts its maximum efficacy against *H*.* pylori* during the bacteria’s reproductive stage. By elevating the stomach’s pH, acid suppressors prompt the bacteria to enter this vulnerable phase while also bolstering amoxicillin’s stability.^9^ Traditional PPIs, however, fall short in maintaining round-the-clock gastric acid control due to their brief half-life, resulting in nocturnal acid breakthroughs for certain patients. Vonoprazan, distinguished by its pKa of 9.06 and belonging to the pyrrole derivative family, accumulates within gastric parietal cells.^32^^,^^33^ Its slow dissociation upon target binding ensures a potent and prolonged acid suppression.^34^ Besides its synergistic pharmacodynamics, the dual therapy with high-dose amoxicillin and vonoprazan offers additional benefits: excellent patient adherence and minimal disruption to the gut microbiota. These advantages have positioned this regimen as a prominent research focus in recent years.^35^^,^^36^

Our research reveals that the eradication rate achieved by this regimen has not met the suggested benchmark. Furthermore, despite achieving an eradication rate comparable to previous regimens and demonstrating a low occurrence of adverse reactions, the first-line eradication therapy for *H*.* pylori* using this regimen requires careful evaluation. This caution is necessitated by significant regional variations in eradication rates and concerns associated with high-dose amoxicillin administration. Therefore, it is imperative to consider local drug resistance patterns, such as the elevated occurrence of adverse reactions and *H*.* pylori* resistance, before implementing this regimen as a first-line treatment.

The subgroup analysis reveals a significantly higher eradication rate of this regimen in China compared to previous treatment programs in the ITT analysis. This discrepancy primarily stems from 2 principal determinants. Firstly, the epidemiological characteristics of *H*.* pylori* play a crucial role. Due to variations in socioeconomic status and lifestyle patterns, the prevalence of *H*.* pylori* infection in China is elevated compared to developed countries.^17^ In terms of drug resistance, *H*.* pylori* strains in China exhibit greater resistance to conventional first-line antibiotics than those in Europe and the United States. Specifically, the resistance rates of *H*.* pylori* to clarithromycin and metronidazole were 22.2% and 69.2%, respectively, in comparison to 34% and 78% in China.^37^ These epidemiological disparities contribute to the diverse therapeutic effectiveness of the same treatment regimen across different geographical regions. Secondly, racial differences also influence the eradication rate. Vonoprazan is primarily metabolized by CYP3A4,^38^ and although previous studies indicate that CYP3A4/5 or CYP2C19 genotypes have negligible influence on the eradication rate of vonoprazan-based regimens,^39^ these investigations were limited to the Japanese population, leaving the impact on European and American populations uncertain.

The present study bears several limitations. Firstly, the epidemiology of *H*.* pylori* exhibits evident regional disparities, and the overrepresentation of Chinese studies in this meta-analysis might introduce a significant geographical bias, thereby potentially impacting the final outcomes. Future investigations encompassing a broader spectrum of geographical regions are warranted for confirmation. Secondly, this analysis solely encompassed patients undergoing primary eradication therapy, therapeutic outcomes in salvage therapy scenarios remain unverified to be further substantiated. Thirdly, numerous factors, including smoking, alcohol consumption, and history of antimicrobial usage, are known to influence the eradication efficacy of *H*.* pylori*. However, due to data constraints, this study did not pursue an in-depth analysis of these variables.

In conclusion, this meta-analysis demonstrated that high-dose dual therapy exhibits non-inferior therapeutic efficacy to quadruple and triple therapies. Furthermore, patients demonstrated a higher willingness to complete the treatment course due to the superior tolerability profile associated with high-dose dual therapy. Additionally, economically advantageous, high-dose dual therapy emerges as a relatively economical option. For patients undergoing initial and rescue treatments, high-dose dual therapy represents an efficacious and safe regimen for *H*.* pylori* eradication. Future research should focus on conducting experiments in diverse regions to systematically investigate geographical variations in treatment responsiveness.

## Supplementary Materials

Supplementary Material

## Figures and Tables

**Table 1.** Baseline Characteristics of the Included Studies

First Author/Year	Country	Duration	Number (H/C)	Regimens		Eradication Rate		AEs
				H	C	ITT	PP	
Haisheng Qian/2022^23^	China	10 days	125/125	VPZ: 20 mg, bid AMO: 750 mg, qid	VPZ: 20 mg, bid AMO: 1.0 g, bid	H: 114/125 (91.2%) C: 103/125 (82.4%)	H: 114/122 (93.4%) C: 103/121 (85.1%)	H: 14/123 (11.4%) C: 10/122 (8.2%)
Haisheng Qian/2022^23^			125/125		ESO: 20 mg, bid AMO: 1.0 g, bid CLA: 500 mg, bid CBP: 200 mg, bid	H: 114/125 (91.2%) C: 110/125 (88.0%)	H: 114/122 (93.4%) C: 110/121 (90.9%)	H: 14/123 (11.4%) C: 29/123(23.6%)
Jie Hu/2023^27^	China	14 days	97/97	VPZ: 20 mg, bid AMO: 1.0 g, tid	ESO: 20 mg, bid MNZ: 400 mg,qid AMO: 1.0 g, bid BPC: 220 mg, bid	H: 86/97 (88.7%) C: 89/97 (91.8%)	H: 86/90 (95.6%) C: 89/92 (96.7%)	H: 15/90(16.7%) C:35/92(38.0%)
William D. Chey/2022^24^	USA or Europe	14 days	349/349	VPZ: 20 mg, bid AMO: 1.0 g, tid	VPZ: 20 mg, bid AMO: 1.0 g, bid CLA: 500 mg, bid	H: 208/265 (78.5%) C: 222/262 (84.7%)	H: 177/218 (81.2%) C: 198/219 (90.4%)	H: 104/348 (29.9%) C: 118/346 (34.1%)
William D. Chey/2022^24^			349/348		LAN: 30 mg, bid AMO: 1.0 g, bid CLA: 500 mg, bid	H: 208/265 (78.5%) C: 201/255 (78.8%)	H: 177/218 (81.2%) C: 174/212 (82.1%)	H: 104/348 (29.9%) C: 119/345 (34.5%)
Xiang Peng/2023^28^	China	14 days	158/158	VPZ: 20 mg, bid AMO: 750 mg, qid	ESO: 20 mg, bid AMO: 1.0 g, bid CLA: 500 mg, bid CBS: 220 mg, bid	H: 142/158 (89.9%) C: 128/158 (81.0%)	H: 142/145 (97.9%) C: 128/141 (90.8%)	H: 30/158 (19.0%) C: 68/158 (43.0%)
Yi Hu/2022^25^	China	7 days	21/24	VPZ: 20 mg, bid AMO: 1.0 g, tid	VPZ: 20 mg, bid AMO: 1.0 g, bid	H: 17/21 (81.0%) C: 16/24 (66.7%)	H: 17/21 (81.0%) C: 16/22 (72.7%)	H: 5/21(23.8%) C: 4/24(16.7%)
Yi Hu/2022^25^		10 days	37/37		VPZ: 20 mg, bid AMO: 1.0 g, bid	H: 30/37 (81.1%) C: 33/37 (89.2%)	H: 30/37 (81.1%) C: 33/37 (89.2%)	H: 9/37 (24.3%) C: 11/37 (29.7%)
Yi Hu/2023^29^	China	14 days	55/55	VPZ: 20 mg, bid AMO: 1.0 g, tid	VPZ: 20 mg, bid AMO: 1.0 g, bid	H: 48/55 (87.3%) C: 49/55 (89.1%)	H: 47/49 (95.9%) C: 48/51 (94.1%)	H: 11/55(20.0%) C: 16/55(29.1%)
Yimin Lin/2022^26^	China	7 days	85/84	VPZ: 20 mg, bid AMO: 750 mg, qid	VPZ: 20 mg, bid AMO: 500 mg,qid	H: 54/85 (63.5%) C: 49/84 (58.3%)	H: 54/83 (65.1%) C: 49/74 (66.2%)	H: - (16.9%) C: - (13.2%)
Yimin Lin/2022^26^			85/61		VPZ: 20 mg, bid AMO: 750 mg, bid CLA: 500 mg, bid	H: 54/85 (63.5%) C: 37/61 (60.7%)	H: 54/83 (65.1%) C: 37/57 (64.9%)	H: - (16.9%) C: - (24.10%)

-, unavailable; AEs, adverse events; AMO, amoxicillin; bid, 2 times a day; BPC, bismuth potassium citrate; C, control group; CBPC, colloidal bismuth pectin capsule; CBS, colloidal bismuth subcitrate; ESO, esomeprazole; H, high-dose amoxicillin and vonoprazan dual therapy; ITT, intention-to-treat analysis; LAN, lansoprazole; MNZ, metronidazole; PP, per-protocol analysis; qid, 4 times a day; tid, 3 times a day; VPZ, vonoprazan.

**Table 2.** Assessment Results of the Risk of Bias

First Author/Year	Randomized Method	Blinding	Allocation Concealment	Incomplete Outcome Data	Selective Reporting	Other Bias
Haisheng Qian/2022^23^	Low risk	Low risk	Low risk	Low risk	Low risk	Low risk
Jie Hu/2023^27^	Low risk	Low risk	Low risk	Low risk	Low risk	Low risk
William D. Chey/2022^24^	Low risk	Low risk	Low risk	Low risk	Low risk	Low risk
Xiang Peng/2023^28^	Low risk	Low risk	Low risk	Low risk	Low risk	Low risk
Yi Hu/2022^25^	Low risk	Low risk	Low risk	Low risk	Low risk	Low risk
Yi Hu/2023^29^	Low risk	Low risk	Low risk	Low risk	Low risk	Low risk
Yimin Lin/2022^26^	Low risk	Low risk	Low risk	Low risk	Low risk	Low risk

**Table 3.** Subgroup Analysis Results of Eradication Rate in Intention-To-Treat and Per-Protocol Analysis

Group Factors	Groups	Number of Studies	OR (95% CI)	Intragroup Heterogeneity		Effect Model	*P*	Intergroup Heterogeneity	
				*P*	*I*^2^ (%)			*P*	*I*^2^ (%)
Study areas (ITT)	China	6^23^^,^^25^^-^^29^	1.34 (1.01, 1.78)	.39	5.0	RE	.04	.1	37.0
	USA or Europe	1^24^	0.81 (0.55, 1.20)	.20	39.0	RE	.29		
Duration (ITT)	7 days	2^25^^,^^26^	1.26 (0.82, 1.95)	.72	0.0	RE	.29	.1	37.0
	10 days	2^23^^,^^25^	1.38 (0.68, 2.79)	.18	42.0	RE	.38		
	14 days	4^24^^,^^27^^-^^29^	0.97 (0.63, 1.47)	.07	53.0	RE	.87		
Study areas (PP)	China	6^23^^,^^25^^-^^29^	1.31 (0.89, 1.93)	.26	21.0	RE	.17	.03	49.0
	USA or Europe	1^24^	0.67 (0.33, 1.35)	.06	72.0	RE	.26		
Duration (PP)	7 days	2^25^^,^^26^	1.03 (0.65, 1.62)	.81	0.0	RE	.91	.03	49.0
	10 days	2^23^^,^^25^	1.38 (0.61, 3.12)	.15	47.0	RE	.44		
	14 days	4^24^^,^^27^^-^^29^	1.02 (0.49, 2.10)	.02	67.0	RE	.96		

IIT, intention-to-treat analysis; OR, odds ratio; PP, per-protocol analysis; RE: random effects model.

**Table 4.** Subgroup Analysis Results of Adverse Event Rate

Group Factors	Groups	Number of Studies	OR (95% CI)	Intragroup Heterogeneity		Effect Model	*P*	Intergroup Heterogeneity	
				*P*	*I^2^* (%)			*P*	*I^2^* (%)
Study areas	China	5^23^^,^^25^^,^^27^^-^^29^	0.56 (0.35, 0.89)	.03	57	RE	.01	.005	64
	USA or Europe	1^24^	0.82 (0.65, 1.02)	.94	0	RE	.08		
Duration	10 days	2^23^^,^^25^	0.74 (0.35, 1.61)	.09	59	RE	.45	.004	66
	14 days	4^24^^,^^27^^-^^29^	0.55 (0.36, 0.85)	.003	75	RE	.007		

OR, odds ratio; RE, random effects model.

## Data Availability

Due to the large number of forest plots of subgroup analysis in this study, they are presented in tabular form in the article. Additional data related to this paper, such as data extraction table and subgroup analysis forest plot, can be obtained by contacting Hongxiu Yu and Zhengwen Zhou.
