# Supplementary material for: First-line Eradication of Helicobacter pylori Infection with High-Dose Amoxicillin and Vonoprazan: A Systematic Review and Meta-analysis
Source: Turk J Gastroenterol. 2025 Jun 16;36(7):410–9. doi: 10.5152/tjg.2025.24371 (PMC12257729; doi:10.5152/tjg.2025.24371)
Supplement: Supplementary Material [file supplementary_material.pdf]

Supplementary File 1. Search strategy

PubMed

#1 amoxicillin[MeSH] OR "high dose amoxicillin"[tiab]

#2 vonoprazan[tiab]

#3 "Helicobacter pylori"[MeSH] OR "H. pylori"[tiab]

#4 #1 AND #2 AND #3

#5 "randomized controlled trial"[pt] OR "clinical trial"[pt]

#6 "eradication rate"[tiab] OR efficacy[tiab] OR safety[tiab] OR randomly[tiab]

#7 #5 OR #6

#8 #4 AND #7

Embase

#1 'amoxicillin'/exp

#2 'high dose amoxicillin':ti,ab,kw

#3 #1 OR #2

#4 'vonoprazan'/exp

#5 'Helicobacter pylori'/exp

#6 'H. pylori':ti,ab,kw

#7 #5 OR #6

#8 'randomized controlled trial'/exp

#9 'clinical trial':ti,ab,kw

#10 'eradication rate':ti,ab,kw

#11 'randomly':ti,ab,kw

#12 'efficacy':ti,ab,kw

#13 'safety':ti,ab,kw

#14 #8 OR #9 OR #10 OR #11 OR #12 OR #13

#15 #3 AND #4 AND #7 AND #14

Cochrane Library

#1 MeSH descriptor: [amoxicillin] explode all trees

#2 (high dose amoxicillin):ti,ab,kw

#3 #1 OR #2

#4 MeSH descriptor: [Helicobacter pylori] explode all trees

#5 (H. pylori) :ti,ab,kw

#6 #4 OR #5

#7 MeSH descriptor: [randomized controlled trial] explode all trees

#8 (clinical trial) :ti,ab,kw

#9 (eradication rate) :ti,ab,kw

#10 (efficacy) :ti,ab,kw

#11 (safety) :ti,ab,kw

#12 #7 OR #8 OR #9 OR #10 OR #11

#13 (vonoprazan) :ti,ab,kw

#14 #3 AND #6 AND #12 AND #13

Web of Science

#1 TS=(amoxicillin OR "high dose amoxicillin")

#2 TS=(vonoprazan)

#3 TS=("Helicobacter pylori" OR H. pylori)

#4 TS=("randomized controlled trial" OR "clinical trial" OR "eradication rate" OR efficacy OR safety OR randomly)

#5 #1 AND #2 AND #3 AND #4

ClinicalTrials.gov

#1 病症或疾病:幽门螺杆菌

#2 干预/治疗:阿莫西林和维诺普拉赞

#3 #1 和 #2
